# Obsessive–Compulsive Symptoms in the General Population Under Stressful Conditions: Lessons Learned from the COVID-19 Pandemic

**DOI:** 10.3390/brainsci14121280

**Published:** 2024-12-19

**Authors:** Luca Pellegrini, Umberto Albert, Claudia Carmassi, Giuseppe Carrà, Francesca Cirulli, Bernardo Dell’Osso, Matteo Di Vincenzo, Mario Luciano, Maria Giulia Nanni, Maurizio Pompili, Gabriele Sani, Alfonso Tortorella, Umberto Volpe, Andrea Fiorillo, Gaia Sampogna

**Affiliations:** 1 Department of Medicine, Surgery and Health Sciences, University of Trieste, 34128 Trieste, Italy; ualbert@units.it; 2 Department of Mental Health, Azienda Sanitaria Universitaria Giuliano Isontina—ASUGI, 34148 Trieste, Italy; 3 School of Life and Medical Sciences, University of Hertfordshire, Hatfield AL10 9EU, UK; 4 Centre for Psychedelic Research, Imperial College London, London SW7 2AZ, UK; 5 Department of Clinical and Experimental Medicine, University of Pisa, 56126 Pisa, Italy; claudia.carmassi@unipi.it; 6 Department of Medicine and Surgery, University of Milan Bicocca, 20126 Milan, Italy; giuseppe.carra@unimib.it; 7 Center for Behavioral Sciences and Mental Health, National Institute of Health, 00161 Rome, Italy; francesca.cirulli@iss.it; 8 Neuroscience Research Center, Department of Biomedical and Clinical Sciences “Luigi Sacco”, University of Milan, 20019 Milan, Italy; bernardo.dellosso@unimi.it; 9 Aldo Ravelli Center for Neurotechnology and Brain Therapeutic, University of Milan, 20122 Milano, Italy; 10 Department of Psychiatry and Behavioural Sciences, Stanford University, Stanford, CA 94305, USA; 11 Department of Psychiatry, University of Campania “L. Vanvitelli”, 80138 Naples, Italy; matteo.divincenzo@unicampania.it (M.D.V.); mario.luciano@unicampania.it (M.L.); andrea.fiorillo@unicampania.it (A.F.); gaia.sampogna@gmail.com (G.S.); 12 Institute of Psychiatry, Department of Biomedical and Specialty Surgical Sciences, University of Ferrara, 44121 Ferrara, Italy; mariagiulia.nanni@unife.it; 13 Department of Neurosciences, Mental Health and Sensory Organs, Faculty of Medicine and Psychology, Sapienza University of Rome, 00185 Rome, Italy; maurizio.pompili@uniroma1.it; 14 Department of Neuroscience, Section of Psychiatry, University Cattolica del Sacro Cuore, 00168 Rome, Italy; gabriele.sani@unicatt.it; 15 Department of Neuroscience, Sensory Organs and Thorax, Fondazione Policlinico A. Gemelli IRCCS, 00136 Rome, Italy; 16 Department of Psychiatry, Fondazione Policlinico A. Gemelli IRCCS, 00136 Rome, Italy; 17 Department of Psychiatry, University of Perugia, 06123 Perugia, Italy; alfonso.tortorella@unipg.it; 18 Clinical Psychiatry Unit, Department of Clinical Neurosciences, Università Politecnica delle Marche, 60121 Ancona, Italy; u.volpe@univpm.it

**Keywords:** OCD, obsessive–compulsive disorder, COVID-19, lockdown, pandemic, COMET, stress factor, trigger

## Abstract

**Introduction**: The COVID-19 pandemic had a negative impact on mental health in the general population. The fear, stress, and uncertainty surrounding that traumatic period could have contributed to the aggravation or possible new onset of obsessive–compulsive symptoms. **Methods:** The COvid Mental hEalth Trial (COMET) is a nationwide project organized by the University of Campania “Luigi Vanvitelli”, designed as an observational investigation that aimed to gather data from a representative sample of the Italian general population. The current study is a report from the main project and it focuses on obsessive–compulsive (OC) symptoms. **Results:** A total sample of N = 20,720 took part in the survey. N = 2332 individuals had a total Obsessive–Compulsive Inventory—Revised (OCI-R) score greater than or equal to 21 (11.3% of the entire sample), indicating the presence of clinically relevant obsessive–compulsive symptoms. By excluding patients with a history of previous mental illnesses, we still obtained a high number of individuals with an OCI-R score greater than or equal to 21 (N = 2024), representing 10.3% of the overall sample, possibly indicating a new incidence of OC symptoms during the pandemic. **Discussion:** Our study highlights a substantial new incidence of obsessive–compulsive symptoms in the general public. Risk factors or red flags such as being male, being of working age, living in a highly stressful environment such as one of the Italian regions most affected and severely hit by the pandemic, having higher levels of loneliness, and using substances to cope with stress, should be paid particular attention in order to prevent the development of OC symptoms during a critical and traumatic event such as the COVID-19 pandemic.

## 1. Introduction

There is now a large amount of evidence that, during COVID-19 pandemic, mental health in the general public worsened [1,2,3]. In a recent study conducted by the COvid Mental hEalth Trial (COMET) network [1], 20,720 participants were recruited from the general public and completed a survey. In this sample, 12.4% of respondents (N = 2555) reported severe or extremely severe levels of depressive symptoms, 17.6% (N = 3627) reported anxiety symptoms and 41.6% (N = 8619) reported feeling at least moderately stressed by the situation; among these three groups, overlapping symptoms such as insomnia (38.8%) and feelings of loneliness (29.5%) were present. The authors found evidence that, while physical isolation and lockdown represent essential public health measures to contain the spread of the COVID-19 pandemic, they are a serious threat to the mental health and well-being of the general population; therefore, as an integral part of the COVID-19 response, mental health needs should be addressed. Moreover, as mental health problems increased, a significant reduction in referrals and self-presentation to community mental health services was reported in Italy [4], with loneliness [5] and bad eating habits [6] being among the most common contributors to mental health issues. A significant psychopathological load associated with COVID-19 was present in healthcare professionals: Sani and colleagues (2022) [7] found that, compared to the rest of the population, healthcare professionals showed a considerably greater risk for mental health illnesses. As previously considered, well-being and mental health in the general population during the COVID-19 pandemic worsened; a meta-review of prevalence including eighteen different meta-analyses found the prevalence of mental health problems to range from 20 to 36% in the general public, with insomnia and stress being the most common issues, with 32.34% (confidence interval (CI): 25.65–39.84) and 36% (CI: 29.31–43.54), respectively [8]. The reasons for the negative impact of the pandemic on mental health might be varied, but it is possible that psychological issues could be a consequence of general stressful environmental triggers, rather than the pandemic and fear of COVID-19 itself. Therefore, it is important to keep trying to investigate the specific psychiatric symptoms in the general public and the possible risk factors behind them, in order to be more knowledgeable and ready to face future critical and stressful events, catastrophes and even possible future pandemics [9].

In this current study, we focus on a specific type of symptomatology during the COVID-19 pandemic in the general public: obsessive–compulsive symptoms (OCS). Obsessive–compulsive disorder (OCD) is a chronic and debilitating condition characterized by a combination of recurrent obsessional thoughts and time-consuming compulsive rituals, often resistant to treatments [10]. OCD is responsible for a significant disability burden globally [11], has a detrimental effect on the quality of life of the individuals affected [12] and can even lead to suicide [13,14]. Studies report 12-month prevalence estimates of 0.7–3.0% in adults and 0.25–0.30% in children [15,16]. A substantial proportion of the population—estimated at 21% and 28% in the studies by Ruscio et al. (2010) [15] and Fineberg et al. (2013) [17]—report subthreshold obsessive–compulsive symptoms that are often accompanied by symptoms of other mental illnesses. It is anticipated that individuals with these illnesses would have difficulty adjusting to new environments and would struggle during stressful events [18,19]. Obsessive–compulsive symptoms have been a significant concern during the COVID-19 pandemic, with various studies highlighting the impact of the pandemic on individuals with obsessive–compulsive disorder [20,21,22,23]. Research indicates that during the early stages of the pandemic, obsessive–compulsive symptoms worsened, particularly for subjects with contamination-related OCD [20]. The prevalence of obsessive–compulsive symptoms throughout this period was investigated by several studies because of the similarity between the contagion-containing measures (such as physical separation, hand washing, mask use, and quarantine) and obsessive–compulsive phenomenology (e.g., contamination worries and frequent washing and/or checking). A systematic review and meta-analysis of 35 studies [24] found that clinically significant OCS were common among the general population during the pandemic: their prevalence was as high as 22% (studies = 19). Specifically, the prevalence of OCS was 36% in pregnant women, 22% in COVID-19 cases, 21% in undergraduate students and 5% in healthcare professionals. However, the heterogeneity in the results was high, given the use of different scales, each with a different cut-off. OCS increased significantly during the pandemic. not only in individuals with a history of the disorder, but also in the ones without such diagnosis [21]. Furthermore, the fear, uncertainty and stress associated with the spread of COVID-19 have not only been linked to the exacerbation of obsessive–compulsive symptoms [25,26], but also associated with the onset of new ones [26]. The impact of the pandemic on obsessive–compulsive symptoms has been observed across different populations, including medical students [27], young adults [28], children and adolescents [23,29,30], and individuals with pre-existing mental illnesses [31]. The increased frequency of contamination obsessions and cleaning compulsions has been a common theme during the pandemic, with limited exposure to COVID-related news potentially serving as a protective factor against symptom deterioration [31]. Moreover, subjects suffering from OCD may encounter significant difficulty in giving up behaviors that they previously believed were essential for safeguarding against COVID-19 infection [19,32,33]. The study conducted by Fineberg et al. (2021) [19] is the sole published research work examining the mental health challenges faced by the general population in response to the relaxation of lockdown measures in the UK. This study took place between July and November 2020, coinciding with the initial phase of easing restrictions. Therefore, the COVID-19 pandemic has had a significant effect on individuals with OCS, leading to the exacerbation of pre-existing symptoms and/or the possible onset of new ones. The fear, insecurity and uncertainty surrounding the pandemic have contributed to the worsening of obsessive–compulsive behaviors, highlighting the need for continued monitoring and support for individuals with OCD during traumatic events such as this one. The COVID-19 pandemic could be adopted as an example or paradigm of a general stressful trigger, affecting the entire population, and possibly eliciting, inducing or exacerbating OCS [34,35,36]. It would be therefore pivotal to know the types and frequencies of OC symptoms in the general public during this period, the possible incidence of new cases of OCD and finally the predictors for this specific symptomatology. The predictors could be particularly important for the future prevention of the exacerbation or new onset of obsessive–compulsive symptoms under traumatic situations and in the context of public stress and trigger factors.

## 2. Materials and Methods

The COvid Mental hEalth Trial (COMET) is a nationwide trial organized by the University of Campania “Luigi Vanvitelli” in collaboration with nine other university sites: Università Politecnica delle Marche, University of Ferrara, University of Milan Bicocca, University of Milan “Statale,” University of Perugia, University of Pisa, Sapienza University of Rome, “Catholic” University of Rome and University of Trieste. The National Institute of Health in Rome’s Center for Behavioral Sciences and Mental Health has supported the dissemination and implementation of the project, in accordance with the clinical guidelines produced by the National Institute of Health, to manage the effects of the COVID-19 pandemic. The COMET study was designed as an observational study that aimed to gather data from a representative sample of the Italian general population. A snowball sampling procedure has been adopted to obtain a large sample of the Italian population and to evaluate the impact of the studied variables on the outcome measures. Snowball sampling is a non-probability sampling method in which new units are recruited by other units to form part of the sample [37].

The whole research procedure may be found in another study [38], and this current study stems from a sub-analysis of the original study sample [38].

Demographic information, such as gender, age, geographical location, employment status and education, as well as clinical information, such as history of physical or mental illnesses and use of illegal substances, have been collected. The Obsessive–Compulsive Inventory—Revised version [39] was used to detect obsessive–compulsive symptomatology; a total score on the OCI-R equal to or greater than 21 indicates clinically significant OCD symptoms. The OCI-R is available in an Italian version with defined normative scores [40]. Other validated and reliable questionnaires included in the study are DASS-21 (Depressive Anxiety and Stress Scale-21) [41]; Impact of Event Scale—short version (IES) [42]; UCLA scale—short version to evaluate levels of perceived loneliness [43]; Suicidal Ideation Attributes Scale (SIDAS) [44]; Severity of Acute Stress Symptoms Adult scale (SASS) [45]; General Health Questionnaire—12 item version (GHQ) [46]; and Coping Orientation to Problems Experienced Inventory—short version (brief-COPE) [47].

This research was carried out in compliance with universally recognized norms of ethical conduct, in alignment with the Declaration of Helsinki and local legislation. The participants granted their written informed permission to participate in this research. The research received approval from the Ethical Review Board of the University of Campania “L. Vanvitelli” (Protocol number: 0007593/i). Informed consent was obtained from all subjects involved in the study.

Quantitative analysis of the data was conducted by using statistical methods. An analysis of descriptive statistics provided a description of the socio-demographics of the overall sample. ANOVA and T-tests for independent samples were used for continuous variables and a chi-square test was conducted for categorical variables to assess differences between groups (different levels of obsessive–compulsive symptomologies). Scores on the OCI-R total and the relative subscales indicating specific OC dimensions were compared between our sample and the Italian norms [40]. Moreover, the prevalence of clinically significant obsessive–compulsive symptoms was estimated, both for the total OCI-R (a score equal to or greater than 21) and for the OCI-R subscales [39]. The sample was subdivided into four different subgroups in order to explore the impact of the pandemic on obsessive–compulsive symptoms in specific populations: individuals affected by COVID-19, healthcare staff, individuals with mental health illnesses (persistent and self-declared) and subjects in quarantine. A multivariate linear regression model, controlled for independent variables such as age, gender, education, occupational status, civil status, other coping strategies, level of social support, having a COVID-19 infection, geographical region and time to exposure to the pandemic, was conducted to determine potential predictors of significant OCD symptomatology. The multiple imputation technique was used to address missing data. Analyses of the data were performed using JASP (Version 0.16.3) [48], a freely available statistical program created by the University of Amsterdam (JASP Team, 2022), and STATA, version 15 [49]. For all analyses, the level of statistical significance was set at *p* < 0.05.

## 3. Results

A total sample of N = 20,720 took part in the survey. N = 2332 individuals had a score equal to or higher than 21 on the OCI-R (11.3%) (indicating the presence of clinically relevant obsessive–compulsive symptoms) [39]. The sociodemographic characteristics of the sample are summarized in Table 1.

The mean OCI-R total score in the entire sample was 10.7 (SD: 8.2). The total score and the scores on the different dimensions of the OCI-R were significantly greater than the Italian normative values (apart from checking and hoarding dimensions; see Table 2). Therefore, in our large population sample, behaviors such as washing, ordering, obsessing and mental neutralizing were significantly more severe compared to normative values.

We then explored the OCI-R scores in the four different subgroups in order to investigate the impact of the pandemic on obsessive–compulsive symptoms in these specific and at-risk populations: individuals affected by COVID-19 (representing a cluster with particular physical and psychological stress), healthcare staff (a cohort under work-related stress), individuals with pre-existing and self-declared mental health illnesses (a vulnerable group), and individuals in quarantine (subjects under psychological stress) (Table 3).

The most affected group in terms of OCI-R severity was the one composed of individuals with mental illnesses; these subjects showed higher scores on all of the items of the OCI-R compared to the normative values. Both groups of individuals affected by COVID-19 and in quarantine had greater scores on the OCI-R than the norms, indicating the impact of stressful events on obsessive–compulsive symptomatology; these two groups had higher severity on all items of the OCI-R apart from the checking dimension, compared to the norms. Healthcare staff had higher scores on the total OCI-R and on the washing dimension only.

We found that N = 2332 individuals had a score higher than or equal to 21 on the OCI-R (11.3%), which is considered significant for indicating the presence of clinically relevant obsessive–compulsive symptoms [39] (Table 4). Such a large percentage implies that around one out of ten individuals from the general public could have experienced clinically relevant obsessive–compulsive symptoms during the pandemic. Individuals with a previous mental illness had higher rates of clinically significant OC symptoms compared to the three other groups. The frequencies of washing and hoarding dimensions were higher in the individuals with a previous mental illness and in individuals affected by COVID-19 (Table 4).

We then compared patients with an OCI-R score greater than or equal to 21 to the ones with a score below 21, and observed that the former group had more depressive, anxious and stress symptoms than the latter. Also, patients with an OCI-R ≥ 21 scored higher in the GHQ score, in the UCLA and in the SASS. No difference was detected in the scores on the SIDAS, measuring attitudes towards suicide, and the Impact of Event Scale—short version, measuring acute and chronic post-traumatic stress disorders (Table 5).

Given that we showed a higher severity of OCI-R scores and higher frequency of clinically significant symptoms in individuals with a previous mental illness, we conducted another analysis by excluding this specific group. We still obtained a significant number of subjects with OCI-R total scores greater than or equal to 21: N = 2024, representing 10.3% of the overall sample. The subsample of people scoring ≥ 21 at OCI-R in individuals without pre-existing mental illnesses was analyzed through multivariate regression analyses to identify possible factors associated with incident OC symptoms (Table 6), and the following independent variables were found to be associated with the new onset of OC symptoms: being in one of the most COVID-19-affected and severely hit Italian regions, an age range from 24 to 54 years, increased levels of perceived loneliness (as measured by the UCLA), male gender and conspicuous use of substances.

## 4. Discussion

Our findings suggest that obsessive–compulsive symptoms are more frequent and more severe in the general population during a critical event such as the COVID-19 pandemic. In particular, people with previous mental illnesses showed higher rates of clinically significant OC symptoms and higher OCI-R scores, and these should be paid particular attention during a stressful situation of this kind. The healthcare professional group had higher scores on the washing dimension compared to the norms; a possible explanation for this could be an overresponse to professional guidelines, which could hypothetically develop as a compulsive behavior if conducted excessively (i.e., the continuous repetition of washing behaviors in the workplace might acquire a compulsive nature). This finding is in line with results from previous studies [50,51].

A significant percentage of our large population sample (11.3%) exhibited clinically relevant symptoms. If we consider the frequency of the specific dimensions of the OCI-R, individuals with previous mental illnesses had higher frequencies in all symptom dimensions than the three other groups. Compared to the individuals without clinically significant symptoms (OCI-R < 21), individuals with an OCI-R greater than or equal to 21 had more depressive, anxious and stress symptoms, experienced more loneliness and had worse general mental health and more severe symptoms of acute stress. Given the highest chance for people with previous mental illnesses to have greater scores on the OCI-R and possibly to have full-blown OCD, we performed another analysis by excluding individuals with a history of mental illnesses (N = 1133) from the total sample (N = 20,720), obtaining a new sample of N = 19,587. Intriguingly, even after excluding the subjects known to have a previous mental illness, the percentage of individuals experiencing significant OC symptomatology remained high (10.3%). This last finding could indicate a possible substantial incidence of new cases of OCD having developed during the pandemic (as high as one out of ten individuals in our sample) and is in line with previous results [19,32]. Then, we tried to answer the question about who is most at risk of developing obsessive–compulsive symptoms (excluding individuals with a previous mental illness) during a critical and stressful event such as the COVID-19 pandemic. We found that living in one of the most COVID-19-affected and severely hit Italian regions, being an adult of working age (24–54 years), being male, being lonely and exhibiting significant use of substances were all independent risk factors. It is important to understand the impact of the pandemic on OC symptoms in the general public because such a traumatic and critical event could happen again in the future, and we can use the COVID-19 pandemic as a paradigm of a stressful environmental trigger [9]. Being a male of working age, living in a highly stressful environment such as one of the Italian regions most affected and severely hit by the pandemic, having higher levels of loneliness and using substances to cope with stress is the profile most at risk of emerging obsessive–compulsive symptoms. These factors could be seen as red flags for the members of the public that are most at risk, under traumatic situations and in the context of general public stress and trigger factors, of developing an OC symptomology; possible preventive strategies could be adopted accordingly [52,53,54].

## 5. Limitations

First, the snowball sampling methodology could have led to a selection bias, with only the individuals who were interested in the psychological and psychiatric consequences of the pandemic deciding to complete the survey, as well as those more confident in using online tools [37].

Second, the cross-sectional nature of the study does not allow us to infer any causal relationship between our study variables. Third, we identified individuals with mental illnesses by asking a direct question about the presence of a pre-existent mental illness, without any further clinical evaluation or use of diagnostic interviews. The same limitation applies to the selection of the COVID-19+ group, which was, again, performed by self-declaration. Lastly, since we were only able to recruit individuals who were at least 18 years old, our sample cannot be said to be completely representative of young people.

## 6. Conclusions

This paper aimed to characterize the obsessive–compulsive symptoms in the general Italian population during the critical event of the COVID-19 pandemic, with the intention to go beyond the mere cross-sectional picture and look at the possible new incidence of clinically significant obsessive–compulsive symptoms and the associated risk factors [55,56,57,58,59,60]. By excluding individuals with a history of mental illnesses, we could hypothesize that subjects scoring higher than or equal to 21 on the OCI-R represented new occurrences of obsessive–compulsive symptoms in the general public. We were then able to point out specific red flags or risk factors that were linked with this new onset. Our sample was large and representative of the general Italian population during the time of the pandemic. In conclusion, we can say that obsessive–compulsive symptoms should be looked at in the general public, especially regarding who is most at risk and which groups could be identified by using the risk factors we found, during traumatic events such as a pandemic, in order to develop tailored preventive and targeted interventions and reduce the long-term consequences of such an impairing condition, both at individual and at population levels [61,62,63,64].

## Figures and Tables

**Table 1:** Sociodemographic characteristics of the global sample.

Variable	Total Sample N = 20,720	OCI-R < 21 N = 18,388	OCI-R ≥ 21 N = 2332	*p*-Value
Gender, female, % (N)	71 (14,720)	70.2 (12,916)	77.4 (1804)	<0.001
Age, mean (SD)	40.4 (14.3)	40.9 (14.3)	36.6 (14.3)	<0.001
Age group, % (N)				<0.001
<24 years	15.2 (3151)	14.0 (2569)	25.0 (582)	
24–55 years	65.2 (13,514)	65.8 (12,104)	60.5 (1410)	
55–65 years	14.0 (2904)	14.5 (2663)	10.3 (241)	
>65 years	5.6 (1150)	5.7 (1055)	4.1 (95)	
Individuals affected by COVID-19, yes % (N)	5.2 (1088)	5.1 (943)	6.2 (145)	0.03
People living in severely hit regions, yes % (N)	31.3 (6485)	31.7 (5828)	28.2 (657)	<0.01
University degree, yes % (N)	62.0 (12,846)	63.1 (11,616)	52.7 (1230)	<0.001
Employed, yes % (N)	70.0 (14,518)	71.4 (13,131)	59.5 (1387)	<0.001
Lost job due to pandemic, yes % (N)	6.3 (1302)	5.8 (1077)	9.6 (225)	<0.001
Marital status, single, yes % (N)	39.1 (8091)	37.5 (6905)	50.9 (1186)	<0.001
Any physical condition, yes % (N)	14.5 (3014)	14.1 (2589)	18.3 (425)	<0.001
Healthcare staff, % (N)	14 (2907)	14.5 (2674)	10 (233)	<0.001
With mental illness, % (N)	5.5 (1133)	4.5 (827)	13.2 (306)	<0.001
In quarantine, % (N)	75 (15,592)	75.8 (13,937)	71.0 (1655)	<0.001

**Table 2:** OCI-R severity in the total sample compared to Italian normative scores.

OCI-R	Total Sample (N = 20,720)	Italian Normative Scores (N = 340) *	*p*-Value	Differences Between the Mean and 95% Confidence Interval
	Mean (SD)	Mean (SD)		
**OCI-R total score**	**10.7 (8.2)**	**7.8 (7.6)**	**<0.001**	**2.9 (2.0 to 3.8)**
OCI-R washing	**2.5 (2.3)**	**0.9 (1.5)**	**<0.001**	**1.6 (1.3 to 1.8)**
OCI-R checking	1.2 (1.7)	1.3 (2.0)	0.28	−0.1 (−0.3 to 0.1)
OCI-R ordering	**2.4 (2.3)**	**1.9 (2.3)**	**<0.001**	**0.5 (0.2 to 0.7)**
OCI-R obsessing	**2.9 (2.6)**	**1.6 (2.4)**	**<0.001**	**1.3 (1.0 to 1.6)**
OCI-R mental neutralizing	**0.5 (1.3)**	**0.3 (0.9)**	**<0.001**	**0.2 (0.1 to 0.3)**
OCI-R hoarding	1.9 (2.1)	1.7 (2.1)	0.81	0.2 (−0.02 to 0.4)

*: Marchetti et al., 2010 [40]. **In bold**: statistically significant differences compared to Italian normative scores (independent sample Student’s *t*-test).

**Table 3:** OCI-R severity in the different groups compared with Italian normative scores.

	Individuals Affected by COVID-19 (N = 1088)	Healthcare Staff (N = 2907)	Individuals with Mental Illnesses (N = 1133)	Individuals in Quarantine (N = 15,592)	Italian Normative Scores (N = 340) ^&^
**OCI-R**	**Mean (SD)**	**Mean (SD)**	**Mean (SD)**	**Mean (SD)**	**Mean (SD)**
OCI-R total score	11.5 (9.0) ***	9.3 (7.5) **	16.1 (11.0) ***	10.6 (8.0) ***	7.8 (7.6)
OCI-R washing	2.7 (2.6) ***	2.6 (2.5) ***	2.9 (2.8) ***	2.5 (2.2) ***	0.9 (1.5)
OCI-R checking	1.3 (1.7)	1.1 (1.5)	1.9 (2.3) ***	1.2 (1.6)	1.3 (2.0)
OCI-R ordering	2.5 (2.4) ***	2.1 (2.2)	3.3 (3.0) ***	2.4 (2.8) ***	1.9 (2.3)
OCI-R mental neutralizing	0.6 (1.5) ***	0.4 (1.1)	1.0 (1.9) ***	0.5 (1.3) *	0.3 (0.9)
OCI-R obsessing	2.4 (2.7) ***	1.6 (2.1)	4.6 (3.4) ***	2.3 (2.5) ***	1.6 (2.4)
OCI-R hoarding	2.1 (2.3) *	1.6 (1.9)	2.6 (2.6) ***	1.9 (2.1)	1.7 (2.1)

**^&^**: Marchetti et al., 2010 [40]. **In bold**: statistically significant differences compared to Italian normative scores (independent sample Student’s *t*-test). ***: *p* < 0.0001; **: *p* < 0.001; *: *p* < 0.01.

**Table 4:** Clinically significant OC symptoms in the total sample and different groups (chi-squared test used for comparisons).

Characteristic	Total Sample	Individuals Affected by COVID-19 (N = 1088)	Healthcare Staff (N = 2907)	Individuals with Mental Illnesses (N = 1133)	Individuals in Quarantine (N = 15,592)	*p*-Value
Clinically significant OCD symptoms (OCI-R ≥ 21)—N (%)	2332 (11.3)	**145 (13.3) ^c^**	**242 (8.3) ^a^**	**308 (27.2) ^a,b,c^**	**1668 (10.7) ^b^**	**<0.001**
Clinically significant washing (score ≥ 5)—N (%)	3279 (15.8)	**215 (19.8) ^b^**	501 (17.3)	**238 (21.0) ^a^**	**2369 (15.2) ^a,b^**	**<0.001**
Clinically significant checking (score ≥ 6)—N (%)	584 (2.8)	**40 (3.7) ^b^**	**74 (2.5) ^c^**	**89 (7.9) ^a,b,c^**	**405 (2.6) ^b^**	**<0.001**
Clinically significant ordering (score ≥ 6)—N (%)	2091 (10.1)	**120 (11) ^c^**	**204 (7) ^b^**	**217 (19) ^a,b,c^**	**1559 (10) ^c^**	**<0.001**
Clinically significant obsessing (score ≥ 8)—N (%)	1210 (5.8)	**80 (7.4) ^a^**	**74 (2.5) ^c^**	**240 (21.2) ^a,b,c^**	**826 (5.3) ^b^**	**<0.001**
Clinically significant mental neutralizing (score ≥ 3)—N (%)	1340 (6.5)	92 (8.5)	**135 (4.6) ^b^**	**153 (13.6) ^a,b^**	**982 (6.3) ^a^**	**<0.001**
Clinically significant hoarding (score ≥ 6)—N (%)	1406 (6.8)	**96 (8.9) ^c^**	**137 (4.7) ^a,c^**	**149 (13.2) ^a,b^**	**1045 (6.7) ^b,c^**	**<0.001**

a,b,c: The letters represent the statistically significant differences between 2 groups (e.g., a with a, b with b, c with c). **In bold**: statistically significant differences between groups.

**Table 5:** Scores from the clinical questionnaires between the groups with OCI-R ≥ 21 and with OCI-R < 21 (Independent Sample Student’s *t*-Test).

	OCI-R < 21		OCI-R ≥ 21		Statistic		
Scale	N	Mean (SD)	N	Mean (SD)	T	*p*-Value	Cohen’s d
DASS-21 Depression	18,388	**11.79 (7.64)**	**2332**	**16.42 (5.22)**	**−28.45**	**<0.001**	**−0.63**
DASS-21 Anxiety	18,388	**6.71 (6.52)**	**2332**	**13.17 (6.30)**	**−45.18**	**<0.001**	**−0.99**
DASS-21 Stress	18,388	**16.13 (7.39)**	**2332**	**17.85 (4.49)**	**−10.97**	**<0.001**	**−0.24**
IES	18,388	6.95 (5.18)	2332	7.14 (5.16)	−1.66	0.01	-0.04
UCLA	18,388	**1.88 (0.54)**	**2332**	**1.92 (0.54)**	**−3.70**	**<0.001**	**−0.08**
SIDAS	4629	4.87 (6.59)	585	4.83 (6.94)	0.14	0.89	0.01
SASS	18,388	**5.19 (4.21)**	**2332**	**12.62 (5.26)**	**−77.94**	**<0.001**	**−1.71**
GHQ	18,388	**17.39 (3.12)**	**2332**	**17.93 (3.11)**	**−7.79**	**<0.001**	**−0.17**

DASS-21: Depressive Anxiety and Stress Scale-21; IES: Impact of Event Scale—short version; UCLA: Revised UCLA Loneliness Scale; SIDAS: Suicidal Ideation Attributes Scale; SASS: Severity of Acute Stress Symptoms—Adult scale; GHQ: General Health Questionnaire—12-item version. **In bold**: statistically significant differences between OCI-R ≥ 21 and OCI-R < 21 groups.

**Table 6:** Predictors of clinically significant OC symptoms (OCI-R score ≥ 21) (excluding individuals with a previous mental health illness).

OCI-R ≥ 21	*p*-Value	Exp(B)	95% C.I.	
			Lower	Upper
**Being in one of the most affected Italian regions**	**0.022**	**1.131**	1.018	1.256
Age groups (reference: up to 24 years)				
**24 to 54 years**	**<0.001**	**1.683**	1.281	2.211
55 to 64 years	0.312	1.137	0.886	1.459
Over 65 years	0.973	0.995	0.755	1.312
**UCLA global score**	**0.025**	**1.019**	1.002	1.035
Time to exposure (reference: week March 30–April 8)				
Week April 15–April 9	0.704	1.076	0.738	1.570
Week April 16–April 22	0.753	1.046	0.788	1.390
Week April 23–April 29	0.799	1.033	0.804	1.328
Week April 30–May 4	0.955	1.005	0.854	1.183
Quarantine	0.836	1.050	0.660	1.671
Mental health professionals	0.481	1.171	0.755	1.815
COVID-19+	0.365	0.830	0.556	1.241
**Gender (reference male)**	**<0.001**	**0.741**	0.664	0.828
COPE: using substance (reference never)				
COPE: using substance sometimes	0.068	1.508	0.970	2.347
**COPE: using substance always**	**0.041**	**1.600**	1.019	2.512
**Constant**	**<0.001**	**0.063**		

**In bold**: statistical significance. Reference: reference used for comparison. Controlled for age, occupational status, civil status, education, other coping strategies, satisfaction with economic condition and level of social support, and weighted for the propensity score.

## Data Availability

Dataset available on request from the authors due to confidentiality.
